# Editorial: Habits: plasticity, learning and freedom

**DOI:** 10.3389/fnhum.2015.00468

**Published:** 2015-08-27

**Authors:** Javier Bernacer, Jose A. Lombo, Jose I. Murillo

**Affiliations:** ^1^Mind-Brain Group, Institute for Culture and Society, University of NavarraPamplona, Spain; ^2^School of Philosophy, Pontifical University of the Holy CrossRome, Italy

**Keywords:** procedural learning, multidisciplinary, habitual, goal-directed, routine

“During much of our waking lives, we act according to our habits, from the time we rise and go through our morning routines until we fall asleep after evening routines. Taken in this way, habits have long attracted the interest of philosophers and psychologists, and they have been alternatively praised and cursed.” This is a passage extracted from a highly cited article by Ann Graybiel, one of the most important researchers on habits in present times (Graybiel, 2008). Indeed, most people agree on considering habits one of the crucial aspects of human behavior. However, although laboratory experiments have contributed to advance our understanding on this issue, the difference between habitual and non-habitual behavior is not clear in real-life conditions. This distinction was established in animal research some decades ago (Dickinson, 1985), but the opposition between goal-directed actions and habits seems to fall short in the case of humans. Are habits definitely rigid, unconscious, automatic, and non-teleological? Motor routines, such as those learnt by a beginner piano player, are one of the main examples of habits in neuroscientific literature. If habits are assigned the four characteristics mentioned above, the relationship between well-learned motor routines and the ability of the experienced pianist to improvise will be rejected. How is it possible that motor routines lead to behavioral plasticity, such as improvisation? Habits allow human agents to release cognitive resources in the performance of well-known actions. This is essential for dual-tasking, as many experiments in neuroscience suggest. Furthermore, it is also useful to enhance the cognitive control of actions, as well as to direct habits to achieve a goal and consciously redress them when needed.

In this collection of articles we discuss the role of habits in human behavioral plasticity, learning, and freedom. To our knowledge, this is the first multidisciplinary approach on habits including contributions from the fields of neuroscience, philosophy, psychology, sociology, computation, history, education, psychiatry, neurology, linguistics, physics, and genetics. If we accept that habits are key elements of human behavior, as Graybiel states, they have to be analyzed from different perspectives. Thus, the opening contributions of this e-book are two multidisciplinary approaches from neuroscience and philosophy, in which we develop in depth our position regarding habits and behavioral plasticity (Bernacer and Murillo, 2014; Lombo and Giménez-Amaya, 2014). We then present a block of articles explaining the notion of habit through history, keeping in mind its influence on contemporary neuroscience. Within this block, Barandiaran and Di Paolo show the evolution of this concept from Aristotle to our days, and explain the development of two trends of thought: the organicist and the associationist (Barandiaran and Di Paolo, 2014). After that, our Research Topic contains a series of articles introducing several philosophers' interpretation of habits: Felix Ravaisson (Gaitan and Castresana, 2014), William James (Blanco, 2014), and Merleau-Ponty (Moya, 2014). Then, we present a set of articles with various views on habits: as configurators of personal identity (Wagner and Northoff, 2014), on their possible role on epigenetics (Novo, 2014), on the relationship between neuroplasticity and a characterization introduced in medieval philosophy (Larrivee and Gini, 2014), and from a dynamic systems perspective (Barrett, 2014). After that, we go deeper into the role of habits in behavioral plasticity from different points of view: a computational model of habits as consolidated patterns of motor behavior (Egbert and Barandiaran, 2014), an anthropological theoretical reflection on their role in the integral development of the person (Güell, 2014), and an explanation of their importance on the social basis of learning (Akrivou and Todorow di San Giorgio, 2014). Since the notion of “cognitive habit” may sound strange in neuroscience, we define it in one of our introductory contributions (Bernacer and Murillo, 2014). The following three articles of this e-book are theoretical and empirical examples of cognitive habits: Pagan-Cánovas and Valenzuela speculate about the possible role of habits in conceptual mapping under the umbrella of blending theory (Cánovas and Manzanares, 2014); also, Sanchez-Cañizares reflect on the role of consciousness in triggering cognitive habits (Sánchez-Cañizares, 2014); and finally, Rashidi-Najbar and collaborators present an empirical study on the importance of reading habits in different tasks (Rashidi-Ranjbar et al., 2014). After this approach on cognitive habits, we include several contributions on their role in human action, again from a multidisciplinary perspective: first, a reflection about the importance of habits as integrators of behavioral “systems” within the agent (Martinez-Valbuena and Bernacer, 2014); second, a computational approach on their importance in model averaging and optimal inference (FitzGerald et al., 2014); third, an opinion article about the relationship between procedural learning and behavioral plasticity (Crespo-Eguílaz et al., 2014); fourth, an experimental research on the link between model-based and model-free explanations of human behavior (Friedel et al., 2014); and finally, a theoretical contribution about the positive role of habits in the treatment of addiction (Güell and Nuñez, 2014). The final block of contributions is a set of four articles about the role of habits in learning: L'Ecuyer introduces her proposal about the importance of wonder in learning (L'Ecuyer, 2014), Balderas summarizes empirical research about the role of habits in learning, even in non-human animals (Balderas, 2014), Orón-Semper relates habits and self-control in adolescent maturation (Orón-Semper, 2014), and we close our Research Topic with an interesting empirical article about the link between episodic and prospective memory (Meier et al., 2014).

In all, we hope that the reader finds this Research Topic encouraging and stimulating for future theoretical and empirical research. We are aware that even the title of our e-book may be considered an oxymoron according to the general view of habits in neuroscience: this is the main reason to propose a multidisciplinary approach on the subject, and to investigate about the importance of habits in our daily lives.

## Conflict of interest statement

The authors declare that the research was conducted in the absence of any commercial or financial relationships that could be construed as a potential conflict of interest.
